# Evaluation of the antiparasitic efficacy of praziquantel against *Prohemistomum vivax* (Cyathocotylidae) metacercariae in naturally infected African catfish (*Clarias gariepinus*)

**DOI:** 10.1038/s41598-026-46340-0

**Published:** 2026-05-20

**Authors:** Mohamed Abdelsalam, Shimaa Abdelkhalek, Reda M. S. Korany, Marwa A. Ibrahim, Dalia A. Abdel-moneam, Mohamad Warda, Azizeh Shadidizaji, Marwa M. Attia

**Affiliations:** 1 Department of Aquatic Animal Medicine and Management, Faculty of Veterinary Medicine, Cairo University, Giza, 12211 Egypt; 2 Department of Pathology, Faculty of Veterinary Medicine, Cairo University, Giza, 12211 Egypt; 3 Department of Pathology, Faculty of Veterinary Medicine, Egyptian Chinese University, Cairo, Egypt; 4 Department of Biochemistry and Molecular Biology, Faculty of Veterinary Medicine, Cairo University, Giza, 12211 Egypt; 5 Department of Physiology, Faculty of Veterinary Medicine, Atatürk University, Erzurum, Turkey; 6 Department of Plant Biotechnology, Faculty of Agriculture, Ataturk University, Erzurum, Turkey; 7 Department of Parasitology, Faculty of Veterinary Medicine, Cairo University, Giza, 12211 Egypt; 8 Department of Parasitology, Faculty of Veterinary Medicine, Egyptian Chinese University, Cairo, Egypt

**Keywords:** Digenetic fish trematodes, Cyathocotylidae, Anthelmintic therapy, Aquaculture therapeutics, Inflammatory gene expression, In silico docking, Diseases, Drug discovery, Immunology, Microbiology

## Abstract

*Prohemistomum vivax* (Cyathocotylidae) is a significant parasitic threat in Egyptian African catfish (*Clarias gariepinus*) aquaculture, causing tissue damage, high prevalence, and economic losses. This study evaluated the dose-dependent efficacy of praziquantel against naturally occurring *P. vivax* encysted metacercariae (EMC) under controlled laboratory conditions and explored its potential mode of action via molecular docking. A total of 105 naturally infected catfish (150–250 g) were randomly assigned to seven groups. Each treatment consisted of three independent replicate tanks (experimental units), with five fish per tank (n = 15 fish per treatment)**.** Treatments included a control group (0 mg/L) and six praziquantel groups administered as single doses (0.5, 2, or 3 mg/L for 24 h) or double doses repeated after 7 days. Parasitological, histopathological, and pro-inflammatory gene expression analyses (TNF-α, IL-1β) were performed. The highest efficacy was achieved with double-dose 3 mg/L, resulting in **9**4.2 ± 2.1% parasite reduction (*p* < 0.001), with clear dose-dependent trends (single-dose: 28.8–67.9%; double-dose: 43.7–94.2%). Histopathological analysis showed reduced cyst burden and tissue lesions, while inflammatory markers were significantly downregulated in treated groups. To investigate praziquantel’s mechanism of action, in silico docking targeted* P. vivax* cytochrome c oxidase subunit I (COI), a key mitochondrial enzyme. Praziquantel exhibited strong binding affinity (–6.1 kcal/mol; RMSD = 0.0 Å), forming stable hydrophobic interactions with conserved residues (Val55A, Ala58A, Leu88A, Phe91A, Trp142A). The localization of Trp142A within the active site suggests potential enzyme inhibition, supporting a mitochondrial mechanism of action. These findings confirm praziquantel’s effectiveness against *P. vivax* EMC and identify COI as a potential molecular target. Given its critical role in human medicine, further research into resistance risks, environmental safety, and regulatory frameworks in aquaculture is recommended.

## Introduction

Egypt is the leading aquaculture producer in Africa and the Middle East and ranks sixth worldwide in total aquaculture output^1^. African catfish (*Clarias gariepinus*) is one of the most important farmed species in Egypt, playing a key role in the country’s food security^2^. However, parasitic diseases pose significant threats to catfish farming, with digenetic trematodes being particularly challenging due to their harmful effects and possible zoonotic risks^3^. *Prohemistomum vivax* (Cyathocotylidae) has emerged as one of the most prevalent digenetic trematodes affecting Egyptian catfish, with recent surveys documenting infection rates reaching 86.6% in commercial farms^4,5^. This parasite follows a complex three-host life cycle that involves piscivorous birds serve as definitive hosts where adult worms produce eggs that are shed into water; miracidia hatch and infect freshwater snails as first intermediate hosts; cercariae emerge from snails and penetrate fish as second intermediate hosts, where they encyst as metacercariae (EMC) in various tissues, particularly skeletal muscle and internal organs^6^. The life cycle completes when infected fish are consumed by piscivorous birds or mammals. The pathogenesis of *P. vivax* infection in catfish is characterized by extensive tissue damage and inflammatory responses. Heavy infections cause muscle fiber atrophy, intermuscular edema, hepatocellular degeneration, and upregulation of pro-inflammatory cytokines including TNF-α and IL-1β^4,5^. These pathological changes might result in reduced growth rates, decreased feed conversion efficiency, and increased mortality, leading to substantial economic losses.

**Fig. 1:**
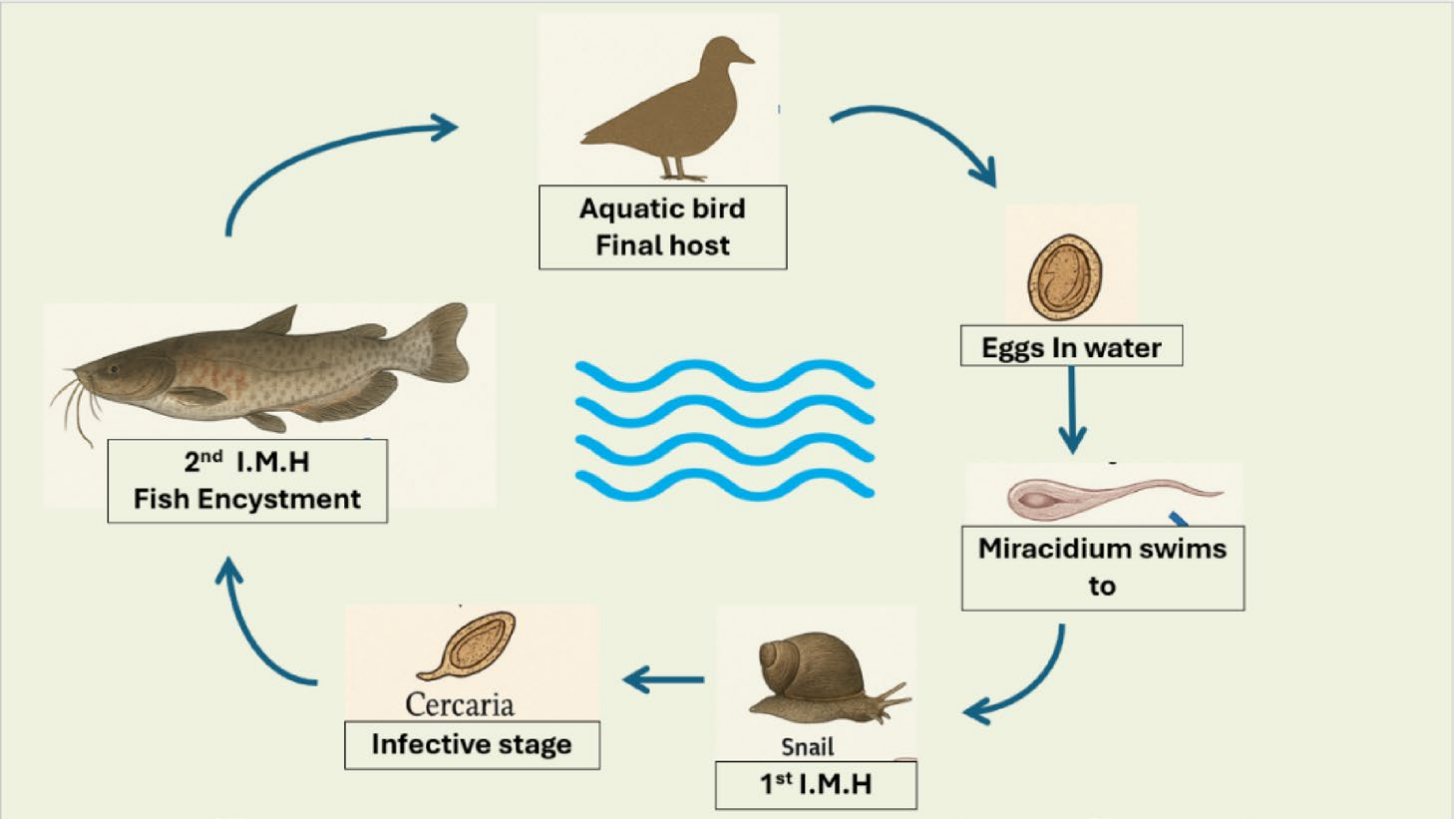
Life cycle of *Prohemistomum vivax* showing host, infective stage, intermediate host and final host.

Current control methods have primarily focused on interrupting the parasite life cycle (Fig. 1) through snail intermediate host management, utilizing either chemical molluscicides (copper sulfate, niclosamide, metaldehyde) or biological control agents such as molluscivorous fish^7,8^. However, these approaches show limited efficacy against encysted metacercariae already present in fish tissues and raise concerns about environmental persistence and impacts on non-target organisms^9^. Traditional chemotherapeutic agents directed against the parasites themselves, including organophosphates (trichlorfon, dichlorvos), benzimidazoles (albendazole, mebendazole), and macrocyclic lactones (ivermectin), demonstrate variable success with narrow safety margins and limited efficacy against EMC^10,11^.

Praziquantel (2-cyclohexylcarbonyl-1,2,3,6,7,11b-hexahydro-4H-pyrazino[2,1-a]isoquinolin-4-one), a synthetic pyrazinoisoquinoline derivative, represents a promising therapeutic candidate due to its highly specific anti-trematode activity and well-established safety profile in both human and veterinary medicine^11,12^. Recent breakthrough research has identified the transient receptor potential melastatin (TRPMPZQ) channel as praziquantel’s primary molecular target, through which the drug causes rapid calcium influx, leading to sustained muscle contraction, tegumental disruption, and parasite death^13^. This mechanism of action, combined with the drug’s ability to penetrate cyst walls^14^, makes it particularly suitable for treating encysted metacercariae.

Praziquantel has demonstrated remarkable efficacy against a wide range of fish trematode parasites across multiple host species^15^. Published studies have reported success rates ranging from 68 to 100% against both adult digeneans and encysted metacercariae, including complete elimination of Clinostomum spp. metacercariae in catfish and effective clearance of Diplostomum spp. in various fish species using both bath and oral treatment regimens^16–20^. The phylogenetic relationship between Diplostomum spp. (Diplostomidae) and Prohemistomum vivax (Cyathocotylidae), both members of the superfamily Diplostomoidea, further suggests potential therapeutic efficacy, given their comparable metacercarial encystment patterns within fish tissues^21^.

Despite this efficacy, the application of praziquantel in aquaculture presents notable challenges. As praziquantel is recommended by the World Health Organization for the treatment of human schistosomiasis, affecting over 240 million people worldwide, its veterinary use must be carefully managed to minimize the risk of accelerating drug resistance development^22,23^. Although resistance to praziquantel remains relatively limited after decades of use, documented cases in human schistosomes and Dipylidium caninum indicate that resistance can emerge under sustained selection pressure^24–26^. In this context, a deeper understanding of the molecular basis of praziquantel’s antiparasitic activity is particularly important. While its primary action is mediated through activation of voltage-gated calcium channels, most notably TRPMPZQ, it has been increasingly recognized that Ca^2^⁺ dysregulation triggers downstream cellular and metabolic stress responses, including effects on mitochondrial function, highlighting the importance of exploring secondary intracellular processes^27^.

Despite these challenges, no published studies have evaluated praziquantel’s therapeutic effectiveness against *P. vivax* EMC in African catfish^3,4^. Given the parasite’s high prevalence in Egyptian aquaculture and the limited efficacy of current control methods, there is an urgent need to assess praziquantel as a potential treatment option in African catfish. Therefore, this study was conducted in a controlled laboratory environment to systematically evaluate the therapeutic efficacy of praziquantel bath treatments against *P. vivax* EMC in naturally infected catfish. The evaluation included parasitological examination, histopathological alterations, and molecular analysis of inflammatory gene expression markers to provide comprehensive assessment of treatment outcomes.

## Materials and methods

### Ethical approval

The study protocol was approved by the Institutional Animal Care and Use Committee of Cairo University (approval number: CU-IACUC-2023-06-15). All procedures followed Egyptian national guidelines for animal research and the Guidelines for the Use of Fishes in Research.

### Fish collection and acclimatization

African catfish (*C. gariepinus*, n = 120, body weight 150–250 g) were collected from a commercial farm in Kafr El-Sheikh Governorate, Egypt (31°06′42"N, 30°56′45"E) where *P. vivax* EMC infection had been previously confirmed^4^. African catfish were transferred alive in plastic buckets supplied with oxygen within the minimum delay time to the wet laboratory for further examinations. Upon arrival, fish were randomly distributed into fiber glass tanks at a stocking density of 5 fish per aquarium in triplicates. Dechlorinated tap water was prepared by 48-h aeration to remove chlorine, under proper aeration conditions (dissolved oxygen > 6 mg/L). Fish were acclimatized for 7 days before treatment initiation. Water parameters were maintained at: temperature 25 ± 1 °C, pH 7.2 ± 0.3, dissolved oxygen > 6 mg/L, total ammonia nitrogen < 0.1 mg/L, nitrite < 0.05 mg/L, and hardness 120–150 mg/L CaCO₃. Photoperiod was set at 12L:12D using timer-controlled fluorescent lighting. Fish were fed commercial pelleted feed (32% crude protein) at 2% of body weight daily during acclimatization. However, fasting began 24 h before treatment and continued throughout the 24-h treatment period. Fish were humanely euthanized prior to sampling using an overdose of tricaine methanesulfonate (MS-222; Sigma-Aldrich, USA) at a concentration of 300 mg/L. The anesthetic solution was buffered with sodium bicarbonate to achieve a neutral pH (7.0–7.5) to minimize distress. Fish were immersed in the solution until complete cessation of opercular movement was observed, followed by confirmation of death through loss of reflexes and absence of response to external stimuli. All procedures were conducted in accordance with institutional ethical guidelines for the care and use of experimental animals.

### Parasitological assessment

#### Morphological identification

Before treatment allocation, 15 fish from the same population were examined to establish baseline infection parameters. Comprehensive parasitological examination was performed immediately post-mortem from standardized tissue samples including muscle, liver, kidney, heart, brain, eyes, gills, and skin. Fresh tissue samples (1 gm) were compressed between two glass plates and examined under stereomicroscope at 10–40 × magnification. All metacercariae in the entire compressed sample were counted and results were expressed as metacercariae per gram tissue. Viability assessment included evaluation of internal structure integrity, presence of excretory granules, response to mechanical stimulation observed as movement within the cyst, and active flame cell activity. Representative specimens were photographed using an Olympus CX41 microscope. Baseline infection intensity was determined^28,29^.

#### Molecular confirmation

Genomic DNA was isolated from individual excysted metacercariae using the QIAamp DNA Mini Kit (Qiagen, Hilden, Germany) following a modified protocol. Single metacercariae were incubated with 90 μL Buffer ATL and 10 μL Proteinase K at 56 °C for 2 h to ensure complete lysis. Subsequently, 100 μL Buffer AL was added and incubated at 70 °C for 10 min, followed by ethanol precipitation and column-based purification according to manufacturer’s instructions. DNA was eluted in 35 μL Buffer AE after 5-min incubation at 37 °C. Quality and concentration were determined spectrophotometrically using NanoDrop ND-1000 (Marshall Scientific, Hampton, USA) before storage at -20 °C. The ribosomal RNA gene cluster (18S-ITS1-5.8S-ITS2-partial 28S) was targeted for species identification using universal primers D1 (5′-AGGAATTCCTGGTAAGTGCAAG-3′) and D2 (5′-CGTTACTGAGGGAATCCTGGT-3′)^30^. PCR amplification employed 25 μL reaction volumes with Maxima® Hot Start PCR Master Mix (Thermo Fisher Scientific) under the following conditions: initial denaturation at 98 °C for 1 min, 40 cycles of 98 °C for 10 s, 56 °C for 30 s, and 72 °C for 2 min, with final extension at 72 °C for 5 min.

Amplified products were gel-purified using Gene JET™ PCR Purification Kit (Thermo Fisher Scientific) and sequenced bidirectionally using Big Dye Terminator v3.1 Cycle Sequencing Kit on an ABI PRISM 3730XL DNA Analyzer (Applied Biosystems). Raw sequences were assembled and edited using BioEdit v7.2.5^31^ to generate consensus sequences. Sequences were compared with GenBank database entries.

#### Experimental design

This study employed a completely randomized design with seven treatment groups to evaluate dose-dependent efficacy of praziquantel. The sample size was determined through a power analysis using G*Power 3.1 software^32^. We allocated 15 fish per group to account for potential losses. Random allocation of fish to treatment groups was performed using computer-generated random numbers.

#### Treatment design

One hundred five naturally infected fish were randomly assigned to seven treatment groups (n = 15 each): untreated control (G1, 0 mg/L praziquantel, ethanol concentration 0.01%), single-dose treatments at 0.5 mg/L (G2), 2 mg/L (G3), and 3 mg/L (G4) praziquantel for 24 h^9,33^, and double-dose treatments at 0.5 mg/L (G5), 2 mg/L (G6), and 3 mg/L (G7) praziquantel for 24 h with 7-day intervals between treatments^15,34^.

Pharmaceutical-grade praziquantel (Biltricide®, Bayer, Germany; 99.9% purity, lot number: PZQ-2023-04) was used for all treatments^9,35^. Stock solution was prepared by dissolving 1 g praziquantel in 50 mL absolute ethanol (analytical grade, 99.9%)^36^, and appropriate volumes were added to treatment tanks to achieve target concentrations, with final ethanol concentration maintained below 0.01% v/v in all tanks^34^.

Praziquantel solution was added to treatment tanks with gentle mixing to ensure uniform distribution. After 24 h of continuous exposure^9^, 100% water exchange was performed with fresh dechlorinated water^33^. For double-dose groups, the second treatment was applied exactly 7 days after the first treatment, following the same protocol^19^. Fish behavior, mortality, and water quality parameters were monitored daily throughout 14-day treatment periods**,** with environmental conditions maintained as during the acclimatization phase.

### Post-treatment evaluation

#### Microscopical examination

At day 14, all surviving fish were euthanized using the protocol described above for comprehensive evaluation. Tissue specimens from liver and dorsal muscle (1 g each) were collected from all fish and processed using the fresh tissue compression method. Metacercariae were classified into three categories: (1) live metacercariae showing intact internal structure with active flame cells and response to mechanical stimulation; (2) dead metacercariae characterized by complete loss of internal structure, absence of excretory activity, and no movement response; and (3) degenerated metacercariae displaying partially disrupted structure with reduced refractility. Treatment efficacy was calculated using the formula: Efficacy (%) = [(Control count—Treated count) / Control count] × 100.

#### Histopathological examination and scoring

Tissue specimens were collected for histopathological examination from 5 fish per group. Standardized samples (5 × 5 × 3 mm) were collected from liver and skeletal muscle. Serial Sects. (5-micron thickness) were prepared from each tissue block. Tissues were fixed in neutral buffered formalin 10% for 24 h, processed through graded alcohols, cleared in xylene, and embedded in paraffin wax. Sections were stained with Hematoxylin and Eosin^37^ and examined by light microscope (Olympus BX50, Japan).

#### Gene expression analysis

Total RNA was extracted from liver and muscle tissues (n = 5 fish per group) using Biospin RNA Extraction Kit (Bioflux, China) following manufacturer’s instructions. RNA quality was assessed using NanoDrop spectrophotometer (Thermo Scientific, USA) with acceptance criteria of A260/A280 ratio > 1.8 and A260/A230 ratio > 2.0. Only high-quality RNA samples meeting these criteria were used for further analysis^38^. CDNA synthesis was performed using reverse transcriptase (Biobasic, Canada) with oligo-dT primers according to manufacturer’s protocol^39^.

Expression levels of pro-inflammatory cytokines TNF-α and IL-1β were quantified using quantitative real-time PCR. Reactions were performed in duplicate using SYBR™ Green qPCR Master Mix (BioEasy) in an iQ™5 iCycler thermal cycler (Bio-Rad, Germany). The cycling protocol consisted of initial denaturation at 95 °C for 3 min, followed by 40 cycles of denaturation at 95 °C for 40 s, annealing at 60 °C for 40 s, and extension at 72 °C for 40 s^40^. Primers were designed based on *C. gariepinus* sequences in GenBank using Primer3 software^41^: TNF-α forward 5’-CCGCTGGTTTCCAACAGTTC-3' and reverse 5’-GAAGTAGAGGCCTTTGCGGA-3' (114 bp; XM_053490102.1); IL-1β forward 5’-AGCAGGCAGGATAAGGTTGT-3' and reverse 5’-ATCACCAGAGAAGGAGTGCC-3' (248 bp; XM_053515722.1); GAPDH forward 5’-CGTTGACGGCCATCCTATCT-3' and reverse 5’-CCACCCTGCAAGTGAGAAGA-3' (141 bp; XM_053487835.1). Gene expression was calculated using the ^2^-ΔΔ^CT method with GAPDH as the reference gene and the untreated control group as calibrator^42^.

#### Computational modeling, molecular docking, and dynamics simulation of praziquantel binding to *prohemistomum vivax* cytochrome C oxidase subunit i

The following in silico analyses were conducted as an exploratory, hypothesis-generating approach and do not imply that cytochrome c oxidase subunit I represents a primary molecular target of praziquantel. To explore potential secondary intracellular interactions that may arise downstream of praziquantel-induced calcium dysregulation, a comprehensive computational workflow was employed. This included ligand and protein structure retrieval, homology modeling, active-site prediction, molecular docking, interaction profiling, and normal mode analysis.

These computational tools were used to assess the theoretical binding feasibility and dynamic behavior of praziquantel in complex with the mitochondrial cytochrome c oxidase subunit I (COI) from *Prohemistomum vivax*. The results are intended to generate mechanistic hypotheses regarding possible mitochondrial-associated interactions rather than experimental validation of drug–target binding.

#### Ligand structure retrieval

The 3D structure of praziquantel (PZQ; PubChem CID: 4891) was obtained from the PubChem database (https://pubchem.ncbi.nlm.nih.gov/ ).

#### Protein sequence retrieval and structure prediction

The partial amino acid sequence of cytochrome *c* oxidase subunit I (COI; mitochondrion-encoded) from *Prohemistomum vivax* (Accession No. UWE21374) was obtained from the NCBI Protein database. Homology-based three-dimensional (3D) structure prediction was performed using the SWISS-MODEL server (https://swissmodel.expasy.org/). The query sequence was aligned against the SWISS-MODEL template library, and the optimal structural template was selected based on sequence identity (> 30%), alignment coverage, and QMEAN scoring metrics. The resulting model was used for downstream molecular docking and dynamics simulations.

#### Preparation of protein structure and praziquantel for molecular docking

Prior to molecular docking, the predicted three-dimensional structure of *Prohemistomum vivax* cytochrome *c* oxidase subunit I (partial, mitochondrion-encoded) underwent structural preprocessing to ensure suitability for docking simulations. Using UCSF Chimera (https://www.rbvi.ucsf.edu/chimera), all non-standard residues and water molecules were removed, polar hydrogens were added to refine protonation states and steric clashes were resolved through energy minimization to optimize the overall protein geometry. Similarly, the 3D structure of praziquantel was prepared using standard ligand preparation protocols, including geometry optimization and energy minimization, to ensure conformational stability and accurate interaction profiling.

#### Active site prediction

The potential active sites of cytochrome c oxidase subunit I (partial, mitochondrion) (*P. vivax*) was predicted using the CASTp Fold web server (https://cfold.bme.uic.edu/castpfold). This computational tool identifies and characterizes binding pockets based on protein structure, providing spatial coordinates for putative ligand-binding regions. The predicted active sites guided subsequent molecular docking studies, ensuring focused analysis on the most probable interaction zones between praziquantel (ligand) and the cytochrome c oxidase subunit I (partial, mitochondrion) (*P. vivax*).

#### Molecular docking

Molecular docking simulations were carried out to evaluate the binding interactions between praziquantel and the predicted structure of cytochrome *c* oxidase subunit I (partial, mitochondrion-encoded) from *P. vivax*. Docking was performed using AutoDock Vina, implemented via the UCSF Chimera interface (https://www.rbvi.ucsf.edu/chimera). Grid box parameters were defined based on active site coordinates predicted by the CASTp Fold server. To ensure comprehensive sampling of the ligand’s conformational space, multiple independent docking runs were conducted. Resulting poses were subjected to clustering analysis to identify predominant binding modes. The top-ranked complexes were assessed based on predicted binding affinity (ΔG, kcal/mol) and key intermolecular interactions, including hydrogen bonds and hydrophobic contacts within the predicted active site residues.

#### Post-docking analysis

Post-docking interaction analysis was performed to characterize the binding profile between praziquantel and *Prohemistomum vivax* cytochrome *c* oxidase subunit I using the Protein–Ligand Interaction Profiler (PLIP) web server (https://plip-tool.biotec.tu-dresden.de/plip-web/plip/index). PLIP automatically identified and categorized non-covalent interactions, including hydrogen bonds, hydrophobic contacts, π–π stacking, and other relevant molecular interactions. This analysis provided detailed spatial and quantitative information on ligand binding modes, enabling comprehensive assessment of docking reliability and interaction specificity. Particular attention was given to residues contributing to binding stability and molecular recognition within the predicted active site.

#### Molecular dynamics analysis

Normal mode analysis (NMA) was conducted using the iMOD server (https://imods.iqf.csic.es) to evaluate the conformational dynamics of the *Prohemistomum vivax* cytochrome *c* oxidase subunit I–praziquantel complex. This approach, based on elastic network modeling, predicts collective molecular motions and flexibility. Key dynamic parameters, including deformation energies, B-factor correlations, and covariance matrices, were analyzed to assess structural stability and identify hinge regions potentially involved in functional movements.

### Statistical analysis

Data were analyzed using SPSS version 25.0 (IBM Corp., Armonk, NY, USA). Normality of the data distribution was evaluated using the Shapiro–Wilk test. Parasite count data were log10-transformed prior to statistical analysis to improve normality and homogeneity of variance. Differences among treatment groups were assessed using one-way analysis of variance (ANOVA) followed by Tukey’s post hoc multiple comparison test. Gene expression data were analyzed using the Kruskal–Wallis test followed by Dunn’s multiple comparison test. Histopathological scores were compared using the Chi-square test. Results are presented as mean ± standard error of the mean (SEM), and statistical significance was considered at *p* < 0.05.

## Results

### Clinical observations and baseline infection parameters

**Fig. 2:**
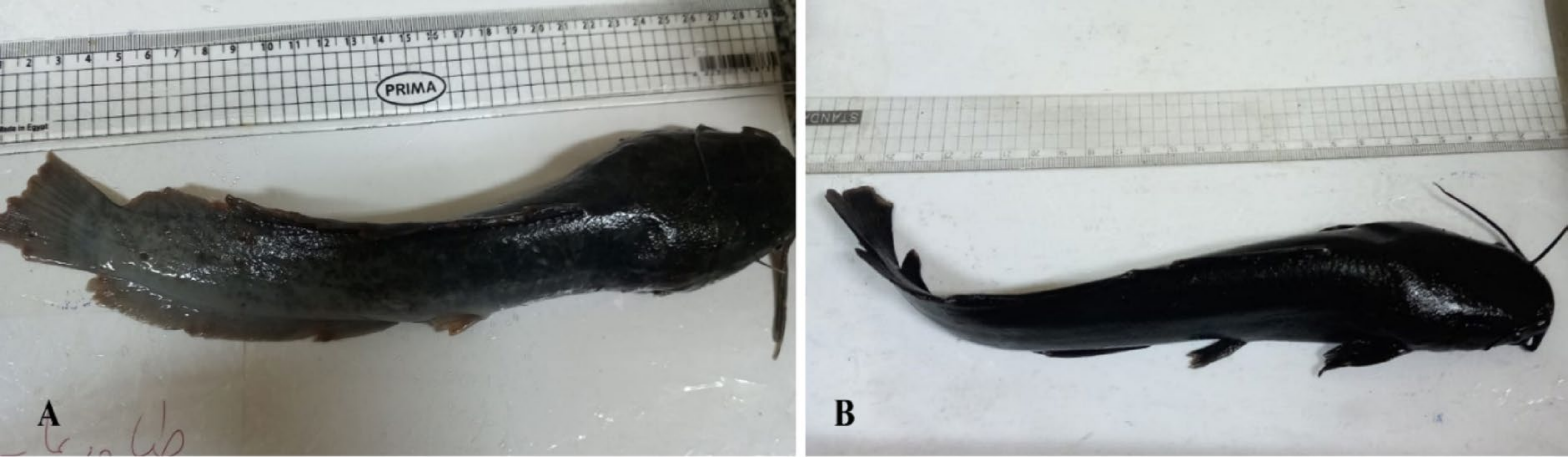
(**A**) Naturally infected African catfish (*Clarias gariepinus*) showing emaciation, excessive mucus production, and frayed, damaged fins. (**B**) Catfish appearance following praziquantel (PZQ) treatment regimens.

Parasitological examination of 15 fish prior to treatment allocation revealed a mean infection intensity of 80 ± 12 encysted metacercariae (EMC) per gram of muscle tissue, with a prevalence of 100% among the examined fish. Parasite counts were determined from standardized muscle samples (1 g tissue) using the fresh compression technique under stereomicroscopy. No significant differences in baseline infection intensity were detected among the experimental groups before treatment allocation (Fig. 2).

### Morphological characterization of *P. vivax* EMC

Microscopic examination revealed that the encysted metacercariae of *Prohemistomum vivax* were oval to spherical in shape and surrounded by a distinct double-layered cyst wall. Detailed morphometric analysis showed that the metacercariae measured 378 ± 11 µm in length (mean ± SD) and 350 ± 6 µm in width (mean ± SD) (n = 50). The cyst wall consisted of two layers: an outer hyaline layer with a mean thickness of 12 ± 2 µm and an inner granular layer with a thickness of approximately 3 ± 0.5 µm. These morphometric characteristics are consistent with previously reported descriptions of *P. vivax* metacercariae and support the taxonomic identification of the parasite.

### Molecular identification

Complete ITS region sequencing from metacercarial specimens produced 1397 bp fragments (GenBank accession: PV583359) encompassing 18S-ITS1-5.8S-ITS2-28S regions. BLAST analysis confirmed taxonomic identity as *P. vivax* (Cyathocotylidae), showing 98.80–99.91% similarity to authenticated *P. vivax* isolates (ON775468, PP747819, OP348897, PP747818, OP348899, OR291421, PQ665119). Lower homology with *Holostephanus* sp. (95.99–85.69%; MT668950, OM755735) and uncharacterized cyathocotylids (84.03–83.87%; PP849714, MN723854) confirmed species-specific identification.

### Efficacy of praziquantel

#### Dose-dependent parasite reduction

Praziquantel treatment led to a significant, dose-dependent reduction in viable *P. vivax* metacercariae across all treatment groups compared to the untreated control (Table 1). The highest efficacy was observed in the double-dose 3 mg/L group (G7), achieving a 94.2 ± 2.1% reduction in parasite burden (95% CI 89.8–97.2%).

**Table 1:** Treatment efficacy of praziquantel against *P. vivax* EMC in African catfish.

Treatment group	Dose (mg/L)	Applications	Post-treatment count*	Reduction from baseline**	% Efficacy***	*p*-value
G1 (Control)	0	–	82 ± 14	+ 2	–	–
G2	0.5	Single	56 ± 8	− 24	28.8 ± 3.2 ^a^	< 0.001
G3	2.0	Single	41 ± 6	− 39	49.6 ± 2.8 ^b^	< 0.001
G4	3.0	Single	26 ± 5	− 54	67.9 ± 3.1 ^c^	< 0.001
G5	0.5	Double	44 ± 7	− 36	43.7 ± 2.9 ^b^	< 0.001
G6	2.0	Double	22 ± 4	− 58	72.7 ± 2.4 ^c^	< 0.001
G7	3.0	Double	5 ± 2	− 75	94.2 ± 2.1 ^d^	< 0.001

*Metacercariae count per gram of muscles (mean ± SEM; n = 15 fish per group).

**Change from baseline mean of 80 metacercariae/g determined from pre-treatment muscle tissues samples.

***Efficacy: different superscript letters indicate statistically significant differences (*p* < 0.05).

Quantitative assessment of parasitic cyst burden revealed a significant reduction in the number of *P. vivax* encysted metacercariae following praziquantel treatment. The mean cyst counts (expressed as mean ± SD per gram of tissue) were significantly lower in treated groups compared with the untreated control. The reduction in cyst burden showed a clear dose-dependent pattern, with higher concentrations and repeated dosing producing greater decreases in cyst numbers. The double-dose 3 mg/L treatment group demonstrated the greatest reduction in cyst counts**,** corresponding to the highest treatment efficacy observed in the study. Statistical analysis confirmed significant differences among treatment groups (*p* < 0.05), supporting the effectiveness of praziquantel in reducing metacercarial cyst burden.

#### Dose–response relationship

Praziquantel efficacy increased proportionally with concentration. Single-dose treatments yielded progressively higher parasite reduction rates: 28.8% at 0.5 mg/L, 49.6% at 2.0 mg/L, and 67.9% at 3.0 mg/L (Table 1). Double-dose regimens produced consistently greater efficacy at each concentration, with the highest reduction (94.2%) achieved at 3.0 mg/L. Statistical analysis confirmed a significant dose-dependent effect across both treatment strategies (*p* < 0.001).

#### Metacercariae viability assessment

**Fig. 3:**
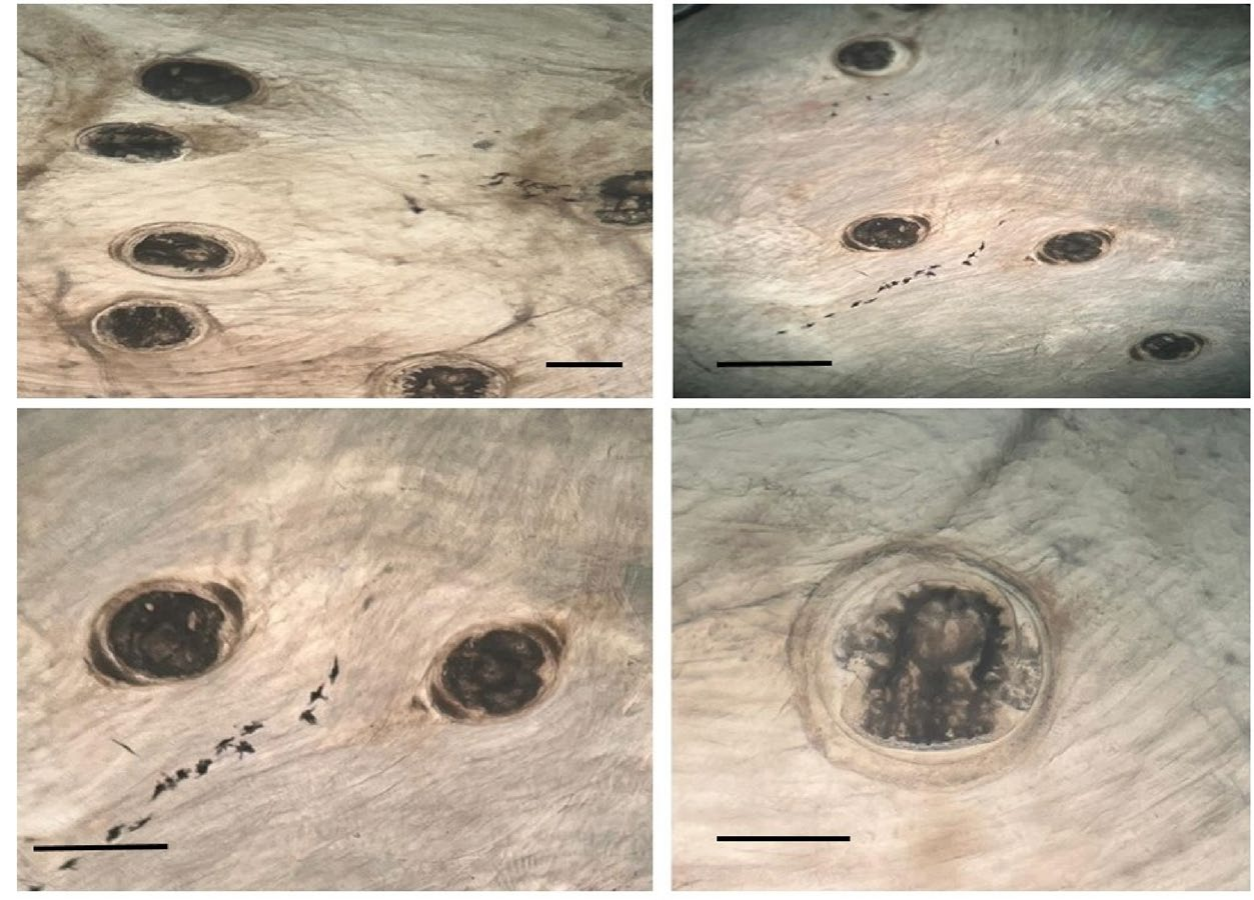
Encysted metacercariae (EMC) of *Prohemistomum vivax* observed at the metacercarial stage; Scale bar: 100 µm.

**Fig. 4:**
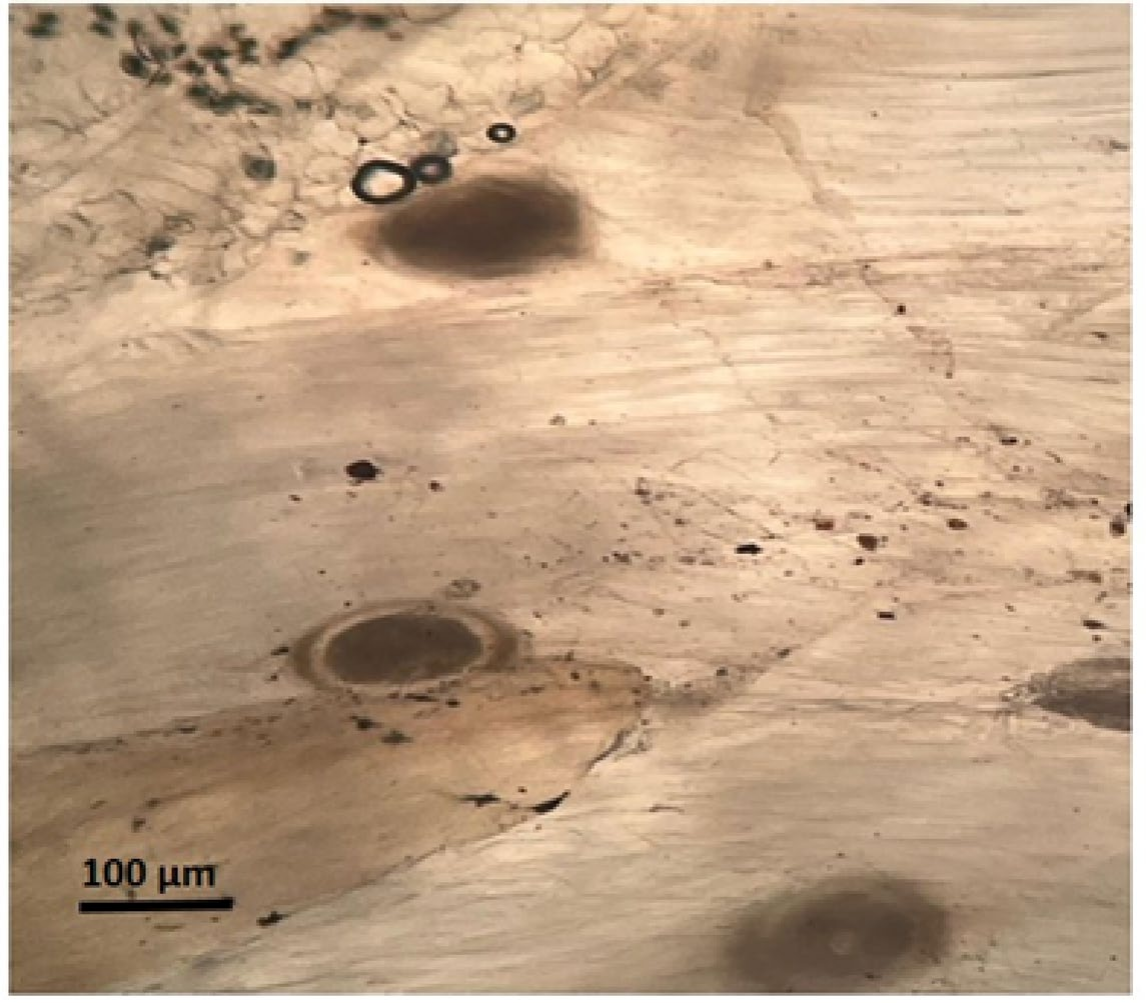
Microscopic examination of tissue specimens from praziquantel-treated fish showing a significant reduction in live encysted metacercariae (*P. vivax*) compared to untreated controls. Note the collapse of metacercarial cysts in wet mount preparations.

Microscopic examination demonstrated a significant increase in the proportion of degenerated *P. vivax* metacercariae following praziquantel treatment (Figs. 3, 4). Quantitative analysis revealed that the percentage of degenerated metacercariae increased in a dose-dependent manner, reaching 16.7 ± 3.2% in the 0.5 mg/L group, 28.6 ± 4.4% in the 2 mg/L group, and 39.2 ± 3.8% in the 3 mg/L single-dose group. Higher degeneration rates were observed in the double-dose treatments, with 31.5 ± 4.1%, 47.6 ± 4.5%, and 53.8 ± 4.2% recorded in the 0.5, 2, and 3 mg/L double-dose groups, respectively. In contrast, only 3.2 ± 1.1% degenerated metacercariae were detected in the untreated control group. Statistical analysis confirmed significant differences among treatment groups (*p* < 0.05); Table 2.

**Table 2:** Viability status of *Prohemistomum vivax* encysted metacercariae (EMC) after praziquantel treatment.

Group	Treatment	Live EMC (%) (Mean ± SD)	Degenerated EMC (%) (Mean ± SD)	Dead EMC (%) (Mean ± SD)
G1	Control (0 mg/L)	95.6 ± 2.4	3.2 ± 1.1	1.2 ± 0.6
G2	0.5 mg/L (single dose)	78.4 ± 4.3	16.7 ± 3.2	4.9 ± 1.4
G3	2 mg/L (single dose)	62.1 ± 5.1	28.6 ± 4.4	9.3 ± 2.1
G4	3 mg/L (single dose)	45.7 ± 4.6	39.2 ± 3.8	15.1 ± 2.7
G5	0.5 mg/L (double dose)	56.3 ± 4.8	31.5 ± 4.1	12.2 ± 2.5
G6	2 mg/L (double dose)	28.9 ± 3.7	47.6 ± 4.5	23.5 ± 3.1
G7	3 mg/L (double dose)	12.4 ± 2.9		

#### Safety assessment

No mortality occurred in any praziquantel-treated group during the 14-day observation period, while the control group experienced 20% mortality (3/15 fish) attributed to heavy parasitic infection. Fish in treated groups resumed normal feeding behaviour within 24–48 h post-treatment, with no observed adverse behavioural effects.

### Histopathological alterations

#### Control group histopathological

**Fig. 5:**
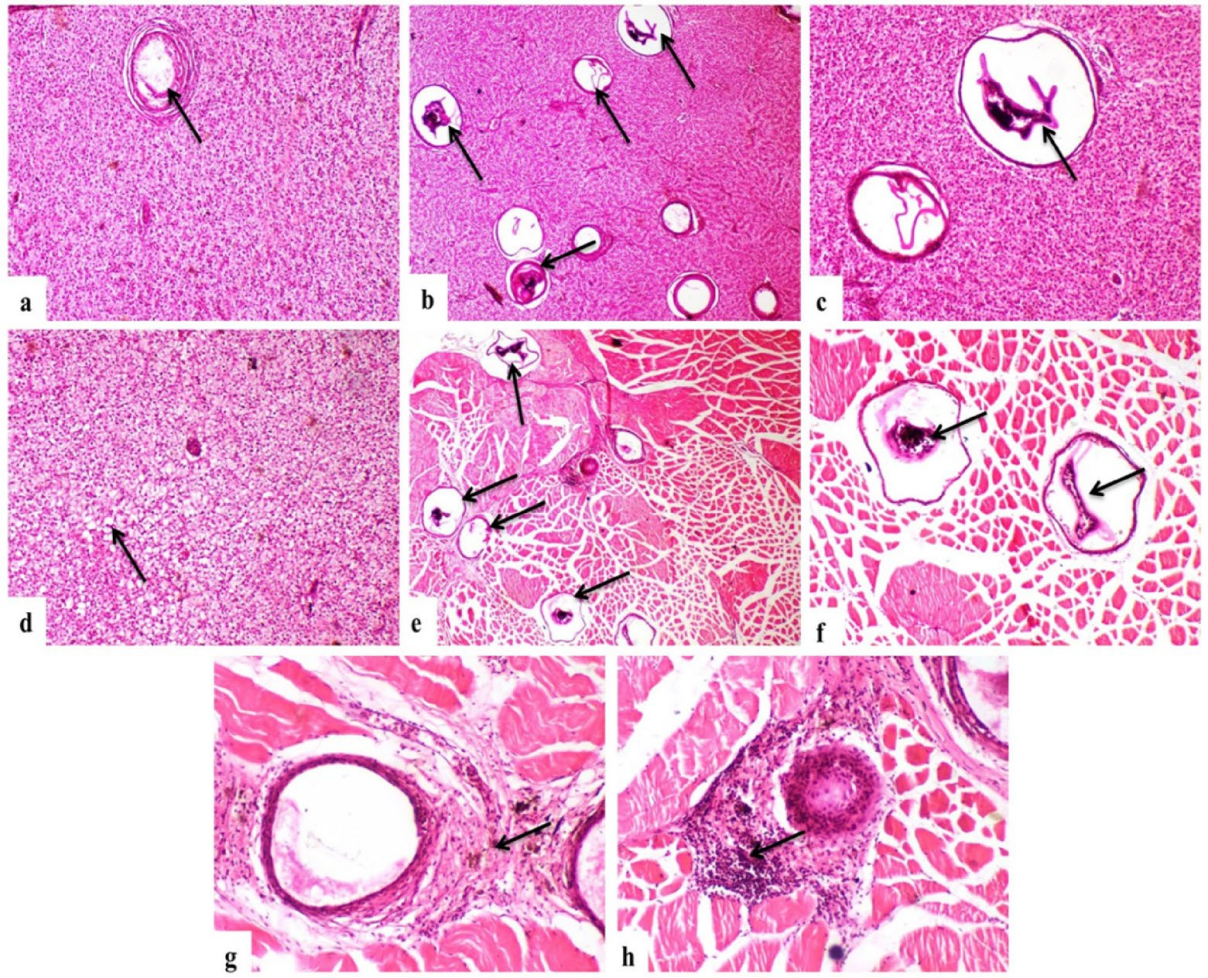
Representative photomicrographs of liver and muscle tissues from untreated African catfish (Group 1, G1) naturally infected with *Prohemistomum vivax* metacercariae. (**a**) A parasitic cyst is observed between hepatocytes (H&E, × 100). (**b**) Multiple cysts are embedded within the liver parenchyma (H&E, × 40), with (**c**) a higher magnification revealing larval stages enclosed within the cysts (H&E, × 100). (**d**) Diffuse vacuolar degeneration of hepatocytes is evident (H&E, × 100). **(e**) Numerous parasitic cysts are present between muscle bundles (H&E, × 40), and (**f**) a closer view illustrates the internal larval stages (H&E, × 100). (**g**) Edema and infiltration of inflammatory cells surround a hepatic cyst (H&E, × 100), while (**h**) heavy inflammatory cell infiltration is observed between muscle fibers (H&E, × 100).

Histopathological examination of control fish (G1) revealed severe tissue alterations associated with metacercariae infection. Liver sections revealed multiple parasitic cysts embedded between hepatocytes (Fig. 5a–c), accompanied by diffuse vacuolar degeneration of hepatocytes (Fig. 5d). Muscle tissues contained multiple parasitic cysts embedded in muscle bundles (Fig. 5e & f ), with observed tissue reaction, inflammatory cells infiltration, edema of interstitial tissue and hyaline degeneration of muscle fibers (Fig. 5 g & h).

#### Treatment-associated improvements

**Fig. 6:**
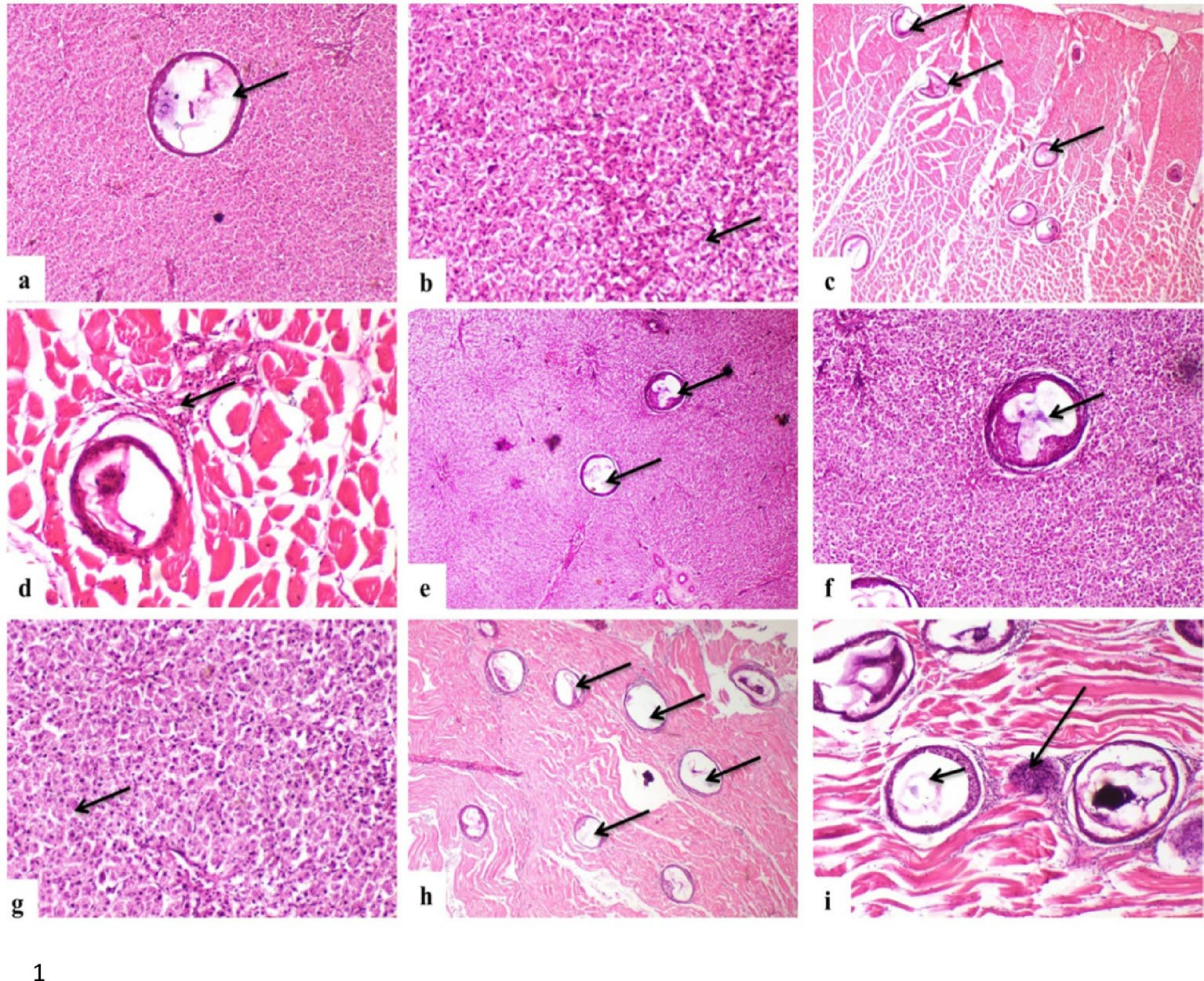
Representative photomicrographs of liver and muscle tissues from praziquantel-treated African catfish in Groups 2 (G2) and 3 (G3). (**a**) G2 showing a parasitic cyst located between hepatocytes (H&E, × 100). (**b**) Moderate hepatocellular vacuolar degeneration is evident in G2 (H&E, × 100). (**c**) Multiple parasitic cysts are embedded within muscle bundles in G2 (H&E, × 40), with (**d**) inflammatory cell infiltration surrounding the cysts (H&E, × 100). (**e**) G3 displaying several parasitic cysts between hepatocytes (H&E, × 40), and (**f**) a higher magnification highlighting degenerated larval stages inside the cysts (H&E, × 100). (**g**) Sporadic hepatocellular necrosis is noted in G3 (H&E, × 100). (**h**) Multiple cysts are also observed within muscle bundles of G3 (H&E, × 40), while (**i**) inflammatory cell infiltration (long arrow) surrounds the cysts (short arrow) (H&E, × 100).

Progressive dose-dependent improvements in histopathological alterations were observed across treatment groups. Single-dose treatments (G2-G4) showed reduced parasitic cyst numbers and decreased inflammatory responses proportional to dose. Groups G2 and G3 retained moderate hepatocellular vacuolar degeneration with fewer parasitic cysts (Fig. 6 a, b, e, f & g), and reduced muscle tissue reaction (Fig. 6 c, d, h & i).

**Fig. 7:**
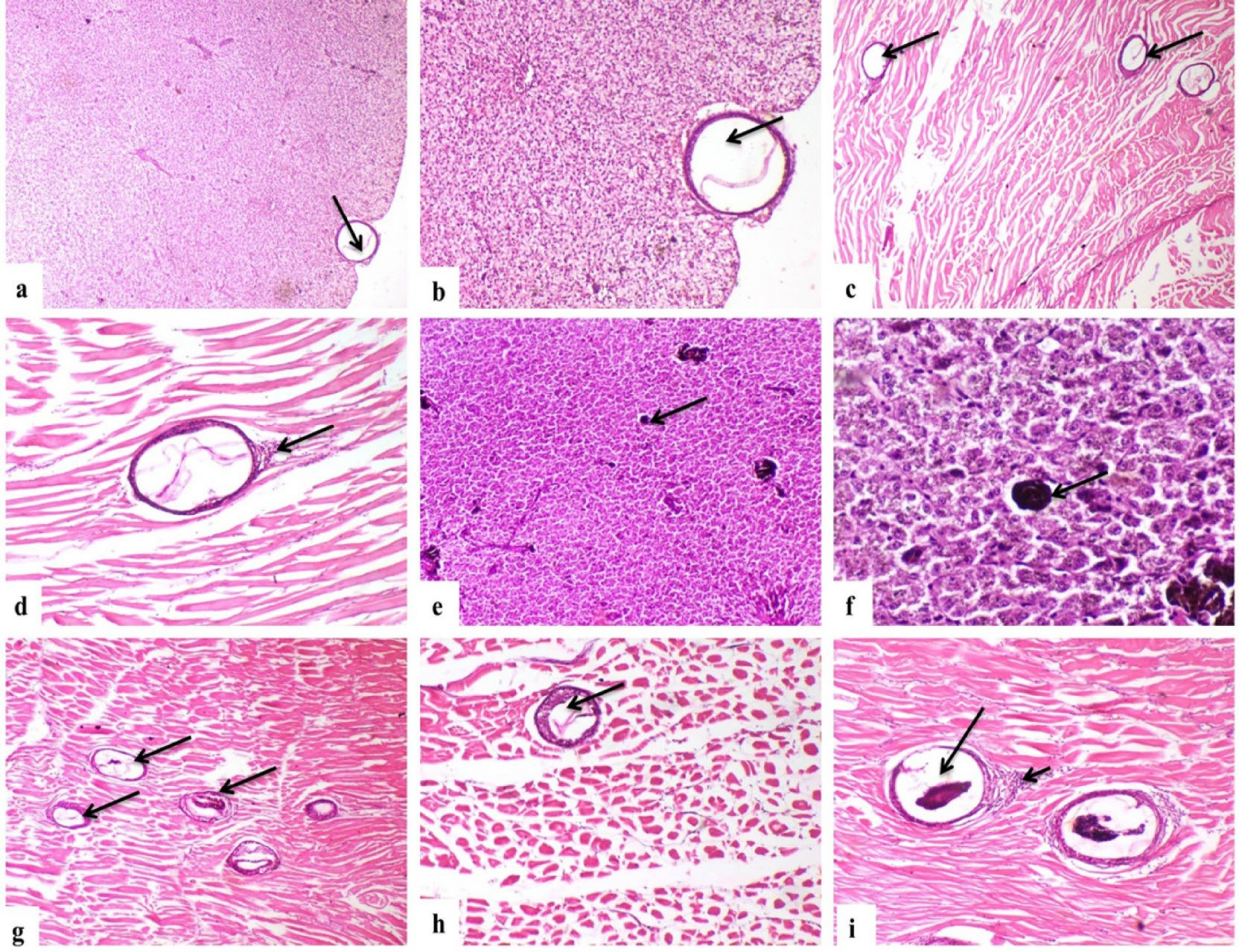
Representative photomicrographs of liver and muscle tissues from praziquantel-treated African catfish in Groups 4 (G4) and 5 (G5). (**a**) G4 shows a parasitic cyst embedded between hepatocytes (H&E, × 40), and (**b**) a higher magnification reveals a degenerated larval stage within the cyst (H&E, × 100). (**c**) Degenerated parasitic cysts are observed within muscle bundles of G4 (H&E, × 40), accompanied by (**d**) mild infiltration of inflammatory cells surrounding the cysts (H&E, × 100). (**e**) In G5, degenerated parasitic cysts are visible between hepatocytes (H&E, × 40), with (**f**) higher magnification confirming cyst degeneration (H&E, × 100). (**g**) Multiple parasitic cysts are seen in the muscle bundles of G5 (H&E, × 40), and (**h**) higher magnification highlights a degenerated larval form inside one of the cysts (H&E, × 100). (**i**) Inflammatory cell infiltration (short arrow) is present around the parasitic cyst (long arrow) in G5 muscle tissue (H&E, × 100).

**Fig. 8:**
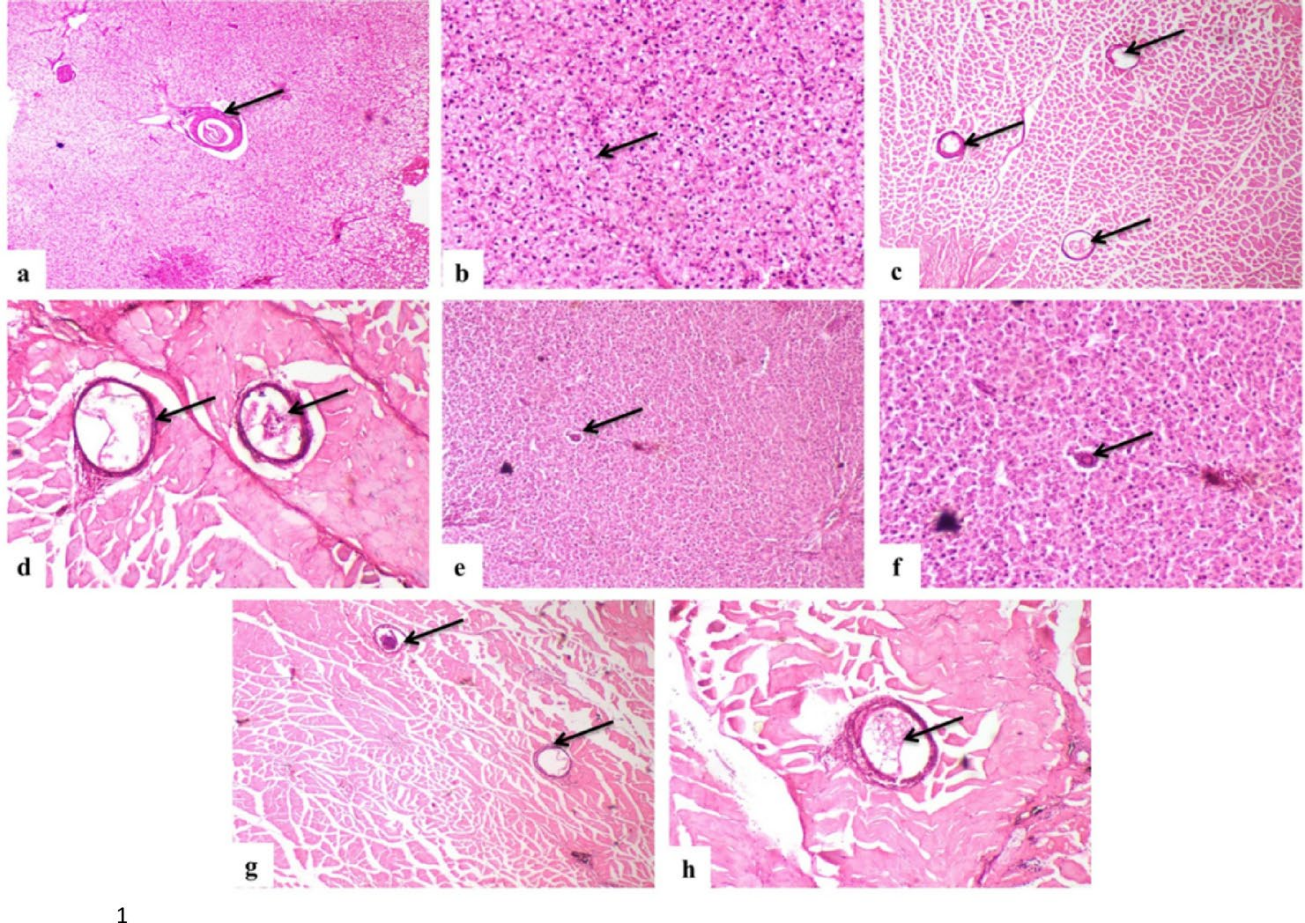
Photomicrographs of liver and muscle tissues from praziquantel-treated African catfish in Groups 6 (G6) and 7 (G7). (**a**) In G6, a parasitic cyst is observed between hepatocytes (H&E, × 40), while (**b**) mild hepatocellular vacuolar degeneration is evident (H&E, × 100). (**c**) Few degenerated parasitic cysts are seen within muscle bundles (H&E, × 40), and (**d**) higher magnification reveals degenerated cysts with no associated inflammatory reaction (H&E, × 100). (**e**) In G7, a degenerated parasitic cyst is present between hepatocytes (H&E, × 40), with (**f**) higher magnification confirming cyst degeneration (H&E, × 100). (**g**) Few degenerated cysts are also visible between muscle bundles (H&E, × 40), and (**h**) degenerated parasitic cysts show no inflammatory response (H&E, × 100).

Higher dose groups (G4, G5) demonstrated marked amelioration with fewer cysts, predominantly degenerated EMC, and mild muscle tissue reaction (Fig. 7a–i). The most effective treatments (G6, G7) revealed minimal pathological changes with rare, degenerated cysts, nearly normal hepatocyte morphology, and minimal inflammatory response in muscle tissue (Fig. 8a–h).

### Inflammatory gene expression analysis

#### TNF-α expression

**Fig. 9:**
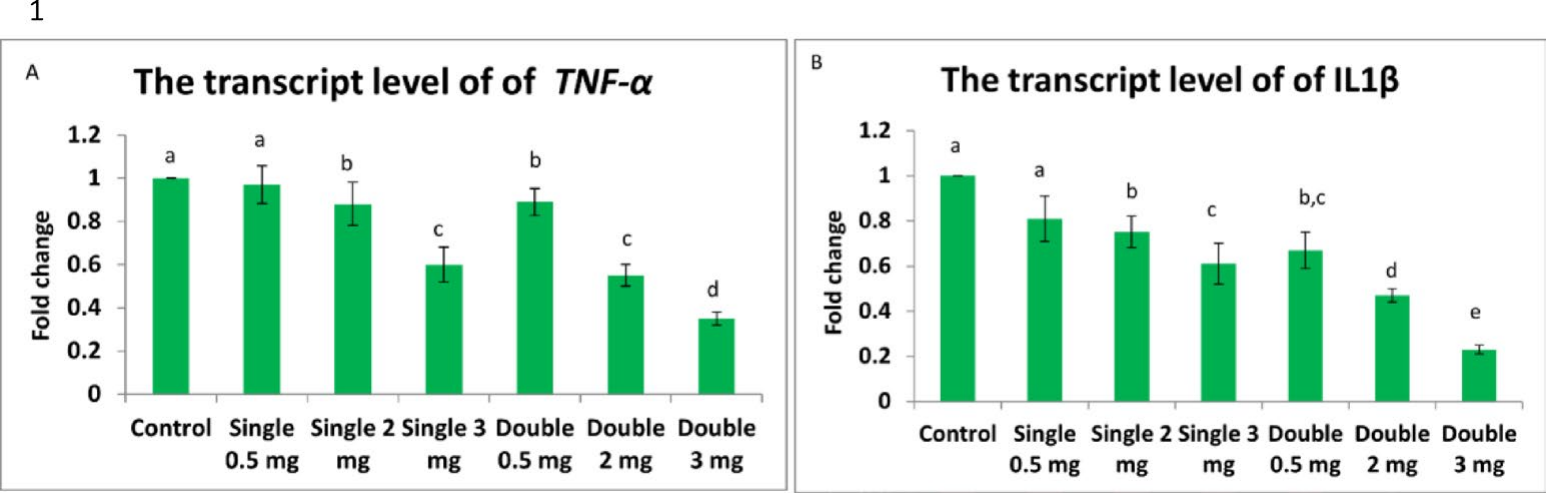
Bar chart illustrating the hepatic transcript levels of (**A**) TNF-α and (**B**) IL-1β in African catfish following praziquantel treatment. Data are presented as mean ± SEM (n = 5 fish per group). Different superscript letters indicate statistically significant differences between groups (*p* < 0.05).

Quantitative RT-PCR analysis revealed significant downregulation of TNF-α expression in both liver and muscle tissues following praziquantel treatment (Fig. 9A, B). In liver tissue, TNF-α expression decreased from baseline (control = 1.0) to 0.72 ± 0.08-fold (G2) through 0.18 ± 0.04-fold (G7), showing clear dose-dependent suppression (*p* < 0.001). Similar patterns occurred in muscle tissue, with expression ranging from 0.78 ± 0.09-fold (G2) to 0.21 ± 0.05-fold (G7).

#### IL-1β expression

**Fig. 10:**
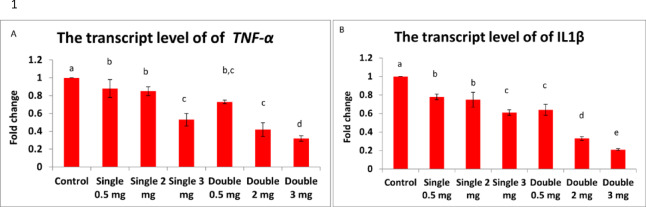
Bar chart showing the transcript levels of (**A**) TNF-α and (**B**) IL-1β in the muscle tissue of African catfish following praziquantel treatment. Values are expressed as mean ± SEM (n = 5 fish per group). Different superscript letters denote statistically significant differences among groups (*p* < 0.05).

IL-1β expression patterns paralleled TNF-α responses, demonstrating dose-dependent downregulation in treated groups (Fig. 10A, B). Liver tissue showed progressive reduction from 0.81 ± 0.07-fold (G2) to 0.15 ± 0.03-fold (G7) compared to controls (*p* < 0.001). Muscle tissue exhibited comparable patterns with final expression levels of 0.19 ± 0.04-fold in G7.

### In silico structural and dynamic characterization of praziquantel binding to *P. vivax* cytochrome *c* oxidase subunit I

#### Molecular docking assessment

The partial amino acid sequence of cytochrome *c* oxidase subunit I (COI; mitochondrion-encoded) from *Prohemistomum vivax* (GenBank Accession: UWE21374.1) was retrieved from the NCBI Protein database. The sequence was as follows: VMFALHLAGDSSSLGSLNFICTIYSCMESLVILRLSVIVWAYLFTSILLLVSLPVLAAAITMLLFDRNFNSAFFDPLGGGDPVLFQHLFWFFGHPEVYVLILPGFGMVSHICLTLSNNDSLFGYFGLVSAMGAIVCLGCIVWAH. A three-dimensional structure of COI was predicted using the SWISS-MODEL homology modeling server. The model selected for docking was based on the highest Global Model Quality Estimation (GMQE) score of 0.95, indicating high structural reliability. Active site prediction was subsequently performed using the CASTp Fold server, which identified a prominent binding pocket (Pocket ID: 2) with a surface area of 3.996 Å^2^ and a volume of 3.874 Å^3^ (Table 3).

**Table 3:** Active site residues of cytochrome *c* oxidase subunit I (COI) from *P. vivax* involved in praziquantel binding.

Chain	Seq ID	AA	ATOM
A	90	TRP	CB
A	90	TRP	CG
A	90	TRP	CD1
A	90	TRP	CD2
A	90	TRP	NE1
A	90	TRP	CE2
A	142	TRP	O
A	144	HIS	O

Residues and atoms from chain an identified using in silico docking as participating in praziquantel binding within the active site of mitochondrial COI. Sequence IDs correspond to amino acid positions in the modeled protein structure.

#### Molecular docking analysis of cytochrome c oxidase subunit 1 with praziquantel

**Fig. 11:**
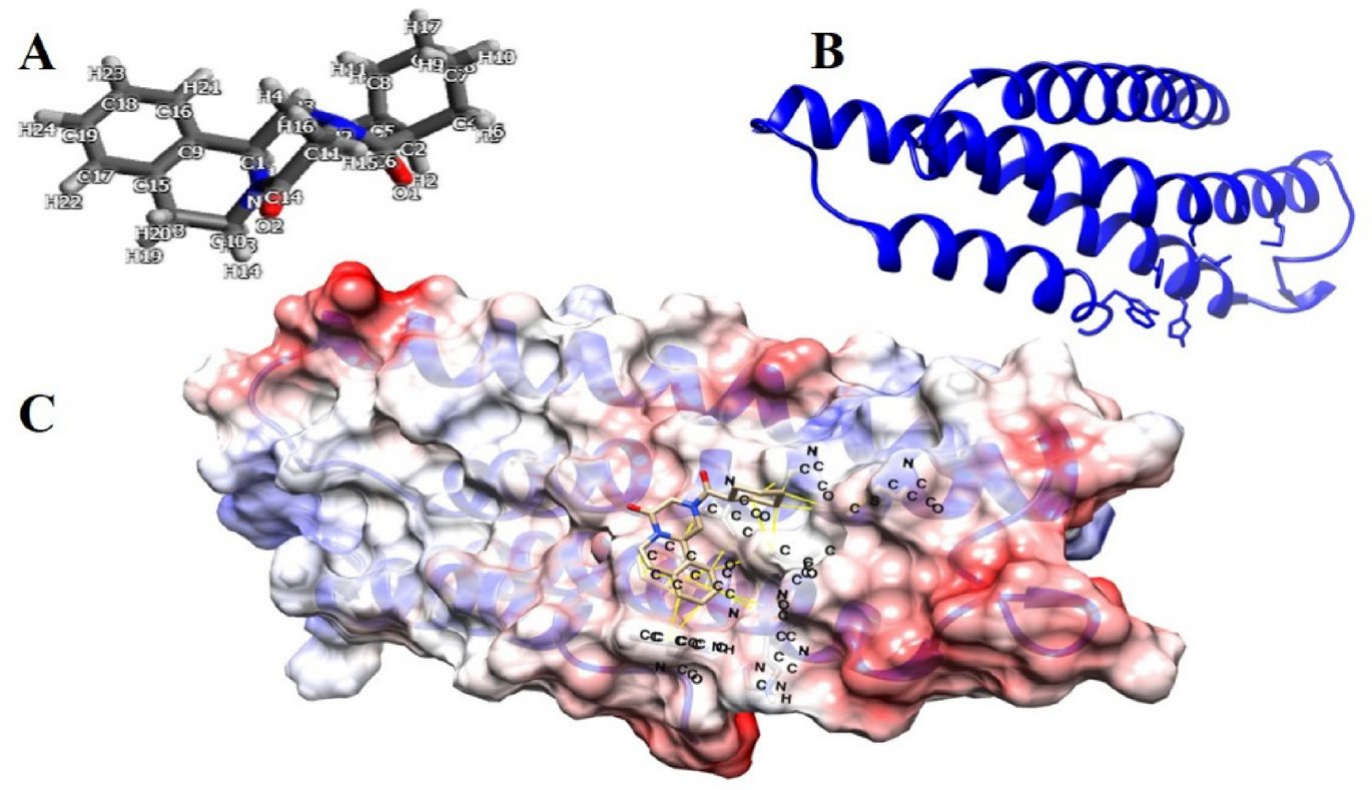
Molecular visualization of praziquantel interaction with *Prohemistomum vivax* cytochrome c oxidase subunit I (COI). (**A**) Structure of praziquantel (PubChem CID: 4891); (**B**) Predicted 3D structure of *P. vivax* cytochrome c oxidase subunit I (mitochondrial); (**C**) Docked complex showing praziquantel bound within the active site of COI.

Molecular docking was conducted to investigate the interaction between praziquantel and the modeled cytochrome c oxidase subunit I (mitochondrion-encoded) from *P. vivax* (Fig. 11A, B). The docking grid was centered at coordinates (12, –10, –5) with dimensions of 20 × 20 × 20 Å^3^, encompassing the predicted active site identified using the Computed Atlas of Surface Topography of Proteins.

Docking simulations revealed a favorable binding interaction, with the top-ranked docking pose (Pose 1) exhibiting a binding affinity score of –6.1 kcal/mol (Fig. 11C). The reported root mean square deviation value of 0.0 Å corresponds to the deviation calculated relative to the top-ranked docking pose, as defined by the docking software output. Because the first-ranked pose is compared against itself, no structural deviation is observed at this reference point, resulting in a root mean square deviation of 0.0 Å. Therefore, this value reflects reference-based structural alignment within the docking procedure rather than experimental structural agreement or time-dependent conformational stability.

To prevent misinterpretation, the root mean square deviation value is considered only within the context of docking pose ranking. The stability and conformational behavior of the protein–ligand complex were further assessed using molecular dynamics simulations and normal mode analysis, as described in the subsequent section.

#### Interaction analysis of praziquantel with *P. vivax* COI using PLIP

**Fig. 12:**
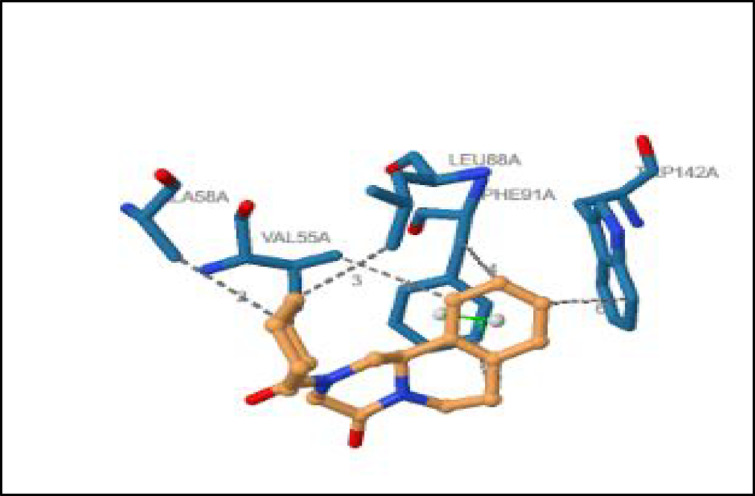
Molecular interaction between *Prohemistomum vivax* cytochrome c oxidase subunit I (COI) and praziquantel. The docking model illustrates key hydrophobic interactions between praziquantel and conserved amino acid residues within the enzyme’s binding pocket, supporting its potential inhibitory effect on mitochondrial function.

Hydrophobic interactions were identified between praziquantel and conserved residues (Val55A, Ala58A, Leu88A, Phe91A, and Trp142A) within cytochrome *c* oxidase subunit I (COI) of *Prohemistomum vivax*, contributing to the stabilization of the ligand within the enzymatic binding pocket. Notably, Trp142A was located within the predicted active site, underscoring its potential role in mediating ligand binding (Fig. 12 and Table 4). Additionally, π–π stacking between praziquantel’s aromatic ring and Phe91A further enhanced binding affinity, potentially contributing to the observed dose-dependent therapeutic efficacy.

**Table 4:** Protein–ligand interaction profile (PLIP) of Prohemistomum vivax cytochrome c oxidase subunit I (COI) and praziquantel.

Index	Residue	AA	Distance	Ligand Atom	Protein Atom
A Hydrophobic Interactions					
1	55A	VAL	3.68	1125	420
2	58A	ALA	3.63	1130	439
3	88A	LEU	3.67	1131	676
4	91A	PHE	3.62	1127	706
5	91A	PHE	3.96	1122	711
6	142A	TRP	3.69	1128	1092

Index	Residue	AA	Distance	Angle	Ligand Atoms
B π-stacking					
1	91A	PHE	4.09	9.64	1119,1124,1125,1126,1127,1128

The PLIP analysis reveals hydrophobic interactions (A) and π-stacking (B) between praziquantel and key residues within the COI binding site, notably involving Trp142A of active site, suggesting stable and specific ligand binding that may interfere with mitochondrial function.

#### Molecular dynamics of the *P. vivax* COI–praziquantel complex (iMOD analysis)

**Fig. 13:**
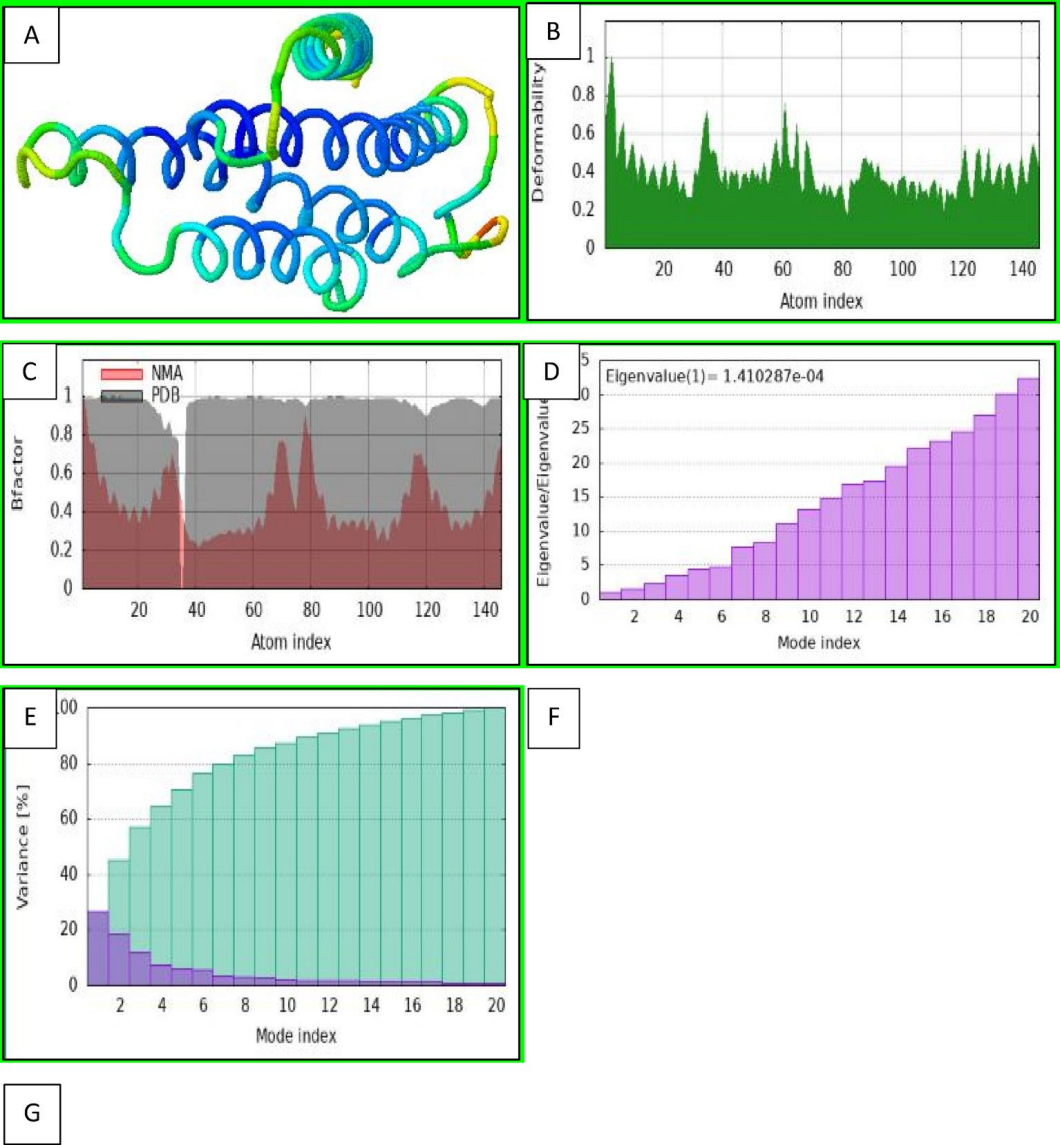
Molecular dynamics simulation results for the *Prohemistomum vivax* cytochrome c oxidase subunit I–praziquantel complex. (**A**) Normal mode analysis (NMA) mobility indicating residue-level flexibility; (**B**) deformability plot showing flexible and rigid regions; (**C**) B-factor values correlating with atomic mobility; (**D**) eigenvalue indicating the energy required for deformation; (**E**) variance plot showing individual (red) and cumulative (green) mode variances; (**F**) covariance map of residue motions, where red indicates correlated, white uncorrelated, and blue anti-correlated movements; and (**G**) elastic network model highlighting stiffer regions in darker gray.

Normal mode analysis (NMA) performed via the iMOD server revealed high flexibility in loop regions, with marked rigidity at the praziquantel-binding site, suggesting a stable complex (Fig. 13A)^43^. Deformability (Fig. 13B) and B-factor plots (Fig. 13C) showed dynamic substrate channels but structural stability around the heme center^44,45^. Eigenvalue (Fig. 13D) and variance analyses (Fig. 13E) indicated dominant low-frequency motions (modes 1–3), reflecting collective functional dynamics^46,47^. Covariance mapping (Fig. 13F) revealed coordinated movements between the ligand-binding pocket and catalytic residues, while the elastic network model (Fig. 13G) highlighted localized rigidity near praziquantel^48,49^. These features support a mechanism by which praziquantel induces conformational stabilization that may interfere with electron transport and proton pumping.

## Discussion

This study presents the first comprehensive evaluation of praziquantel bath treatment efficacy against *Prohemistomum vivax* metacercariae in African catfish (*C. gariepinus*), demonstrating significant dose-dependent therapeutic effectiveness ranging from 28.8% at the lowest concentration (0.5 mg/L, single dose) to 94.2% at the highest concentration (3 mg/L, double dose). These findings align closely with established efficacy patterns documented across related trematode species and position praziquantel as a strong candidate for managing cyathocotylid infections in aquaculture systems.

The molecular docking analysis conducted in this study should be interpreted as a hypothesis-generating computational approach rather than definitive evidence of direct molecular targeting. While the docking results suggested a potential interaction between praziquantel and cytochrome c oxidase subunit I (COI) of *P. vivax*, these findings remain predictive in nature and require further experimental validation. Additional functional and biochemical studies, including enzyme inhibition assays, mitochondrial activity analyses, and in vitro binding experiments, will be necessary to confirm the biological relevance of the predicted interaction. Therefore, the in silico findings should be interpreted with appropriate caution, and future studies are needed to experimentally verify the proposed molecular mechanisms underlying praziquantel activity against *P. vivax*.

The observed dose–response—ranging from 28.8% reduction at a single 0.5 mg/L dose to 94.2% at a double 3 mg/L dose—is consistent with previous reports on praziquantel’s effectiveness against other digenetic trematodes. For instance, treatment achieved 68–100% efficacy against *Clinostomum marginatum* in catfish^16,17^ and similar outcomes have been documented against *Diplostomum spathaceum* in teleosts^18,19^**.** These findings suggest a conserved mechanism of action across trematode taxa.

Supporting this, molecular docking simulations revealed that praziquantel binds strongly to cytochrome *c* oxidase subunit I (COI) of *P. vivax* (binding affinity = –6.1 kcal/mol), a mitochondrial enzyme vital for parasite respiration. The drug formed hydrophobic interactions with conserved residues (Val55A, Ala58A, Leu88A, Phe91A, Trp142A), with Trp142A located at the active site, potentially impairing COI function and thus parasite energy metabolism. The stable binding pose (RMSD = 0.0 Å) aligns with praziquantel’s mechanism of disrupting essential flatworm proteins^13^. These in silico findings reinforce the in vivo efficacy results, implying that praziquantel’s effect may be mediated through mitochondrial dysfunction. Further support came from molecular dynamics simulations using the iMOD server, which demonstrated low deformability and high structural stability at the binding interface, suggesting sustained inhibitory potential.

In light of the observed in vivo efficacy, the in silico analyses were undertaken as an exploratory assessment of whether praziquantel could theoretically interact with cytochrome c oxidase subunit I (COI), a mitochondrial enzyme essential for parasite respiration. Molecular docking predicted a plausible binding pose of praziquantel within the modeled COI structure (binding affinity =  − 6.1 kcal/mol), characterized predominantly by hydrophobic interactions with conserved residues (Val55A, Ala58A, Leu88A, Phe91A, and Trp142A), including Trp142A positioned near the predicted active site. Subsequent molecular dynamics and normal mode analyses indicated limited conformational flexibility and overall structural stability at the protein–ligand interface under simulated conditions, suggesting that the predicted complex could be maintained dynamically. Collectively, these computational observations are consistent with prior reports indicating that praziquantel-induced Ca^2^⁺ influx can trigger downstream cellular and metabolic stress responses in flatworms^13,27^.

Importantly, these findings should be interpreted with appropriate caution. The interaction between praziquantel and COI was evaluated exclusively using in silico approaches, which provide hypothesis-generating insights but do not constitute experimental confirmation of direct drug–target binding or enzymatic inhibition. Accordingly, cytochrome c oxidase subunit I is not proposed as a primary molecular target of praziquantel, whose principal mechanism of action is mediated through activation of voltage-gated calcium channels, particularly TRPMPZQ^13,27^. Rather, the computational results suggest a potential secondary mitochondrial-associated interaction that may arise downstream of praziquantel-induced Ca^2^⁺ dysregulation. Experimental validation through biochemical assays, mitochondrial functional studies, and enzyme inhibition analyses will be necessary to confirm this interaction and to clarify its possible contribution to praziquantel’s antiparasitic activity in future investigations.

Despite this efficacy, low-level infection persistence (5.8%) at the highest dose raises control concerns. This aligns with prior observations that metacercarial cyst walls can hinder drug penetration^50^. Indeed, the hyaline outer layer (~ 12 ± 2 μm thick) in *P. vivax* likely limits praziquantel access, necessitating repeated dosing or higher concentrations to achieve complete clearance.

Morphological changes post-treatment, including cyst wall rupture, internal collapse, and flame cell inactivity, mirrored damage reported in *Schistosoma* and *Clonorchis*^51–53^, reflecting praziquantel’s action on voltage-gated calcium channels, including TRPM PZQ^13^. Histopathological recovery by day 14—marked by resolution of lesions and inflammatory infiltration—paralleled downregulation of TNF-α and IL-1β, confirming an association between parasite clearance and immunomodulation^54^.

From a practical standpoint, implementing praziquantel treatment in Egyptian aquaculture raises regulatory and economic challenges. The absence of local withdrawal periods necessitates adoption of international benchmarks. The EU’s 300 degree-day withdrawal guideline^55^ offers a provisional standard. Estimated treatment costs ($3.50–5.00 USD/m^3^) are significant but offset by reduced mortality and growth suppression. With untreated controls showing 10% mortality and infected fish experiencing 25–40% growth loss^3,4^, treatment appears cost-effective.

Given praziquantel’s global significance in human medicine, especially for schistosomiasis affecting over 240 million people^22^, judicious veterinary use is essential. Documented resistance in *Schistosoma mansoni*^25,56^ and reduced efficacy against *Dipylidium caninum*^26^ highlight resistance risks. To mitigate this, we recommend restricting use to confirmed infections above 50 metacercariae/g tissue, based on our data showing clinical signs above 80/g. This aligns with responsible use principles and avoids unnecessary drug application.

While praziquantel remains a key therapeutic tool, sustainable parasite control will require integrated management strategies. The complex life cycle of *P. vivax* provides multiple intervention points beyond chemotherapy. Snail control via habitat modification, biological agents, or molluscicides can disrupt cercarial stages^7,57^. Preventing bird access using netting or deterrents blocks definitive host input, with proven success in farm settings^58,59^. These environmental interventions, coupled with routine pond monitoring, can reduce drug dependence^60^.

Limitations of this study include the controlled lab environment, which may not fully reflect field complexities such as water quality, co-infections, and nutrition. The 14-day observation period, while suitable for short-term efficacy, cannot detect long-term recrudescence or reinfection^53^. Furthermore, praziquantel pharmacokinetics in *C. gariepinus* remain poorly understood. Studies on drug absorption, distribution, metabolism, and excretion are needed to refine treatment protocols and establish residue-based withdrawal periods.

In light of these findings, we advocate for developing a national regulatory framework for praziquantel use in Egyptian aquaculture. This should include approved protocols (dose, duration, method), mandatory withdrawal times based on pharmacokinetic data, treatment documentation, and veterinary oversight. Aligning with international standards will safeguard consumer safety and praziquantel efficacy for both veterinary and human applications.

## Conclusion

Praziquantel bath treatment at a double 3 mg/L dose achieves 94.2% efficacy against *P. vivax* metacercariae in African catfish with no adverse effects. Molecular docking and dynamics simulations revealed stable binding to cytochrome *c* oxidase subunit I, supporting a mitochondrial mechanism of action that aligns with observed in vivo efficacy. To preserve drug effectiveness, treatment should be limited to confirmed infections above 50 metacercariae/g tissue, guided by mandatory withdrawal periods and veterinary supervision. Integrating chemotherapy with preventive strategies targeting intermediate and definitive hosts can reduce reliance on praziquantel. Establishing a national regulatory framework is essential to ensure responsible use while safeguarding this critical antiparasitic agent for both veterinary and human health applications.

## Data Availability

No datasets were generated or analysed during the current study. Competing interests The authors declare no competing interests.
